# Development and Properties of Rapid-Hardening and High-Fluidity UHPC-Based Grout with Sulfoaluminate Cement and Wollastonite Fibers

**DOI:** 10.3390/ma19143051

**Published:** 2026-07-15

**Authors:** Peipeng Li, Yanbo Wang, Feiyang Li, Xinyi Ran

**Affiliations:** School of Civil Engineering and Architecture, Wuhan University of Technology, Wuhan 430070, China

**Keywords:** UHPC grout, sulfoaluminate cement, wollastonite fiber, rapid hardening, high fluidity

## Abstract

This study develops a rapid-hardening and high-fluidity ultra-high-performance cement (UHPC)-based grout by incorporating calcium sulfoaluminate (CSA) cement as an early strength component, along with steel and wollastonite fibers as hybrid reinforcements. The UHPC grout proportions containing different contents of CSA cement and wollastonite fibers were designed to investigate fluidity, mechanical properties, hydration kinetics, microstructure and chloride resistance. The results showed that both CSA cement and wollastonite fibers significantly enhanced the compressive and flexural strength of UHPC grout. The incorporation of CSA cement led to rapid compressive strengths of 23 MPa at 6 h and 75.9 MPa at 1 day, marking a significant enhancement compared to the reference group and indicating excellent early-age performance. CSA cement accelerated the hydration process of the UHPC grout and promoted formation of more ettringite. Wollastonite fibers and U-type expansive agent (UEA) further improved the mechanical performance through bridging and physical filling effects. Moreover, CSA cement and wollastonite fibers effectively optimized expansion behavior and refined pore structure of the UHPC grout, and improved its chloride penetration resistance. Although both components influenced the fluidity of the grout, the UHPC grout still maintained high fluidity, offering a promising outlook for its potential use in demanding engineering applications.

## 1. Introduction

With the continuous advancement of infrastructures such as marine structures, wind turbine foundations, and long-span bridges, high-performance building materials are significantly needed to achieve excellent strength and service life, especially in extreme conditions [1]. As a result, the development of cement-based materials with excellent mechanical properties, compactness, and durability is essential for enhancing the performance of marine structures. In particular, potential applications such as joint filling and structural reinforcement impose higher requirements on grouting materials. These materials are expected to possess high fluidity and achieve rapid hardening at early ages, while simultaneously maintaining adequate long-term strength and resistance to corrosion, thereby ensuring long-term durability and mechanical performance under complex conditions [2].

Cement-based grout is an inorganic slurry with high fluidity, using cement as the primary binder [3]. Cement-based grout is widely recognized for offering excellent fluidity, self-compacting capability, early-age strength, expansion behavior, and high durability. The good mechanical and durable properties of cement-based grout make it essential in modern civil engineering. However, with increasingly demanding engineering conditions, the performance of conventional cement-based grouts is no longer sufficient to satisfy evolving structural requirements.

Ultra-high-performance concrete (UHPC) is a cement-based material with exceptional mechanical strength and durability. Traditional UHPC typically incorporates large amounts of supplementary cementitious materials (SCMs) to reduce cost and carbon emissions [4,5]. However, such modifications often lead to inadequate early-age strength, particularly under low-temperature conditions. In addition, insufficient workability limits the application of UHPC in crack repair, structural reinforcement, and rapid construction scenarios [6]. Previous studies also indicate that the balance between initial flowability and strength development is a key design issue for grouting materials [7]. In some recent studies, the UHPC examined exhibited relatively low fluidity (<260 mm), and the addition of early-strength components further reduced fluidity by 15% to 40% [8,9]. Current research on UHPC has primarily focused on enhancing the early strength of UHPC, while systematic investigations into the comprehensive optimization of early-age performance, fluidity, long-term strength development and durability remain scarce. Multi-objective considerations have been emphasized in some sustainable geopolymer composites research, where proportion changes may improve reaction degree and strength development but simultaneously affect flowability and setting behavior [10]. Conversely, CSA cement would potentially improve grout strength, especially within 24 h, and microfibers such as wollastonite fibers could provide reinforcement on a microscale.

**Figure 1:**
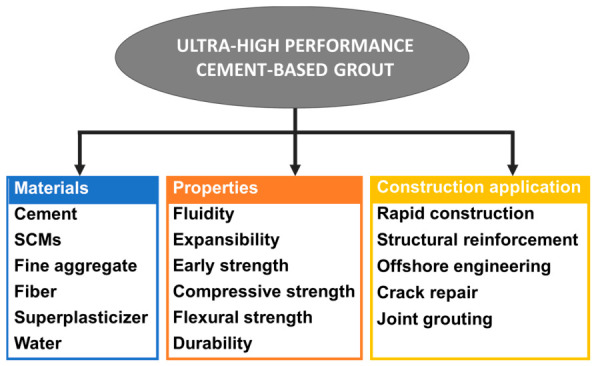
Ultra-high-performance cement-based grout system.

UHPC grout combines the high strength and durability of UHPC with the excellent workability of grouting materials, thereby exhibiting promising application prospects in various engineering fields. Compared to conventional UHPC, it is suitable for a wider range of construction. In addition to its rapid-hardening property, UHPC grout also reduces cracks induced by shrinkage, thereby offering superior durability. To illustrate its full potential, the materials, key properties, and representative construction application scenarios of UHPC grout are systematically presented in Figure 1.

Ordinary Portland Cement (OPC) is the most commonly used binder in UHPC. However, the production of OPC inevitably involves high energy consumption and substantial carbon dioxide emissions. Researchers have explored partially replacing Portland cement with a variety of SCM to address this issue [11,12]. Recent studies on blended cement systems have also demonstrated that sustainable mixture design should not be limited solely to reducing cement consumption. The balance among chemical activity, filler effects, hydration products, and pore refinement is crucial for optimizing mechanical properties [13]. Another effective strategy for reducing the carbon footprint of cement production is to develop eco-friendly cement based on minerals that require a lower amount of limestone, such as calcium sulfoaluminate (CSA) cement. CSA cement is a type of cement primarily produced from limestone and bauxite. Ye’elimite ($C_{4}A_{3}\bar{S}$) is one of the primary mineral phases in CSA cement, which reacts in the early stages of hydration and develops high early strength. These characteristics indicate that CSA cement has the potential to serve engineering applications such as crack repair, joint grouting, structural reinforcement, and rapid construction. Besides its outstanding properties, CSA clinker also has lower direct carbon dioxide emissions compared to OPC clinker. Furthermore, the combination of OPC and CSA cement can integrate the advantages of both systems while allowing for control over specific properties such as fluidity, expansion, and early strength. Meanwhile, considering that CSA cement may experience flash setting in low water-binder ratio systems, the dosage of CSA cement must be selected with caution, and preliminary experiments are highly necessary [14].

Wollastonite is a natural mineral primarily composed of SiO_2_ and CaO [15]. Wollastonite fibers possess a needle-like morphology with a high aspect ratio, a Mohs hardness of 4.5, a density of approximately 2.9 g/cm^3^, a tensile strength of 2700–4100 MPa, and an elastic modulus ranging from 303 to 530 GPa [16]. Studies have shown that the incorporation of wollastonite fibers can significantly improve the early compressive strength of UHPC [17]. These fibers enhance the fracture toughness, flexural strength, and tensile strength of cementitious composites [18]. Moreover, when used in combination with SCMs such as fly ash, wollastonite can reduce the porosity of concrete and enhance its durability [19]. As an inert material [17,18], wollastonite fibers generally remain chemically inactive within the cement matrix. Due to their wide range of sources, wollastonite fibers can be obtained from natural minerals or produced synthetically. These studies indicate that wollastonite fibers not only enhance the mechanical properties of cement-based materials but also offer environmental benefits, making them a high potential reinforcing material [15].

In this study, CSA cement was used as a partial replacement for OPC to prepare ultra-high-performance cement-based grout (UHPC grout). Based on this proportion, steel-wollastonite fibers and U-type expansive agent (UEA) were incorporated. The spread flow, vertical expansion rate, compressive and flexural strength, hydration heat, pore structure, chloride penetration resistance, hydration products, and microstructure of the UHPC grout were systematically investigated.

## 2. Experimental Program

### 2.1. Raw Materials

**Figure 2:**
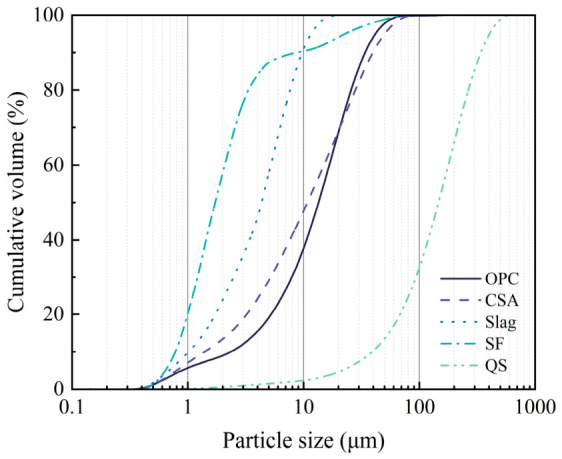
Particle size distributions of binders and quartz sand.

**Figure 3:**
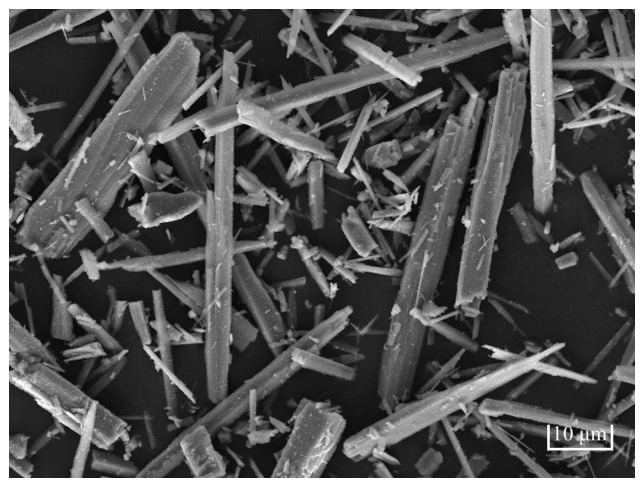
The microstructural morphology of the wollastonite fibers.

The raw materials include PI 52.5 OPC, CSA cement, silica fume (SF), slag powder, quartz sand (QS), water (W), polycarboxylate superplasticizer (SP), U-type expansive agent (UEA, mainly composed of calcium sulfoaluminate and CaO), steel fiber and wollastonite fiber (WF). The chemical compositions of the binders determined by X-ray fluorescence spectrometry are shown in Table 1. The particle size distribution determined by laser diffraction is shown in Figure 2. The wollastonite fibers exhibit an aspect ratio of 15–20:1. Meanwhile, Figure 3 illustrates the microstructural morphology of the wollastonite fibers.

### 2.2. Mixture Design and Sample Preparation

#### 2.2.1. Mixture Design

According to our previous studies [4,20], in the REF group, OPC, silica fume, and slag accounted for 65%, 5%, and 30% of the total binder mass, respectively. The sand-to-binder ratio is 0.8. The water-to-binder ratio is 0.18. To maintain a low water-to-binder ratio and improve fluidity, a polycarboxylate superplasticizer at 0.6% by binder mass was used. Steel fibers were incorporated at a volume fraction of 2%. CSA cement was incorporated at 5%, 10%, and 15% of the total cement content based on preliminary trials and a literature review, thereby enhancing its early-age mechanical performance. Wollastonite fibers were added at volume fractions of 0%, 2%, and 4%. In the presence of wollastonite fibers, an expansive agent was simultaneously incorporated at 3% of the total binder mass to improve the expansion behavior of the UHPC grout. The proportions of mixtures are presented in Table 2.

#### 2.2.2. Preparation Process of UHPC Grout

**Figure 4:**
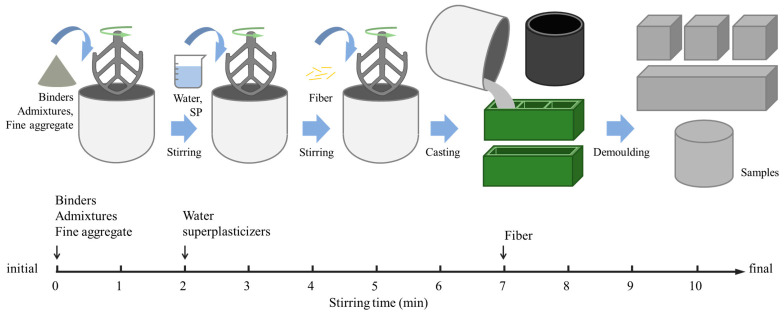
Mixing procedures.

First, the blended powders and quartz sand were poured into the mixer and stirred for 2 min to ensure uniform mixing. Then, the pre-measured water and polycarboxylate superplasticizer were combined and added steadily into the mixer using a beaker, followed by continuous stirring for 5 min to obtain a slurry. Finally, steel fibers and wollastonite fibers were added, and stirred for 3 min until the UHPC grout reached a homogeneous state. The detailed mixing procedures are shown in Figure 4.

### 2.3. Experimental Testing

#### 2.3.1. Fluidity Test

The spread flow of pastes was measured by using a truncated cone (height 60 mm, top diameter 70 mm, bottom diameter 100 mm), in accordance with GB/T 2419-2005 [21]. The fresh paste was poured into the flow cone mold, which was then lifted vertically to allow the grout to flow freely without vibration. The fluidity was determined for each group as the average of two perpendicular diameters of the spread flow.

#### 2.3.2. Vertical Expansion Test

**Figure 5:**
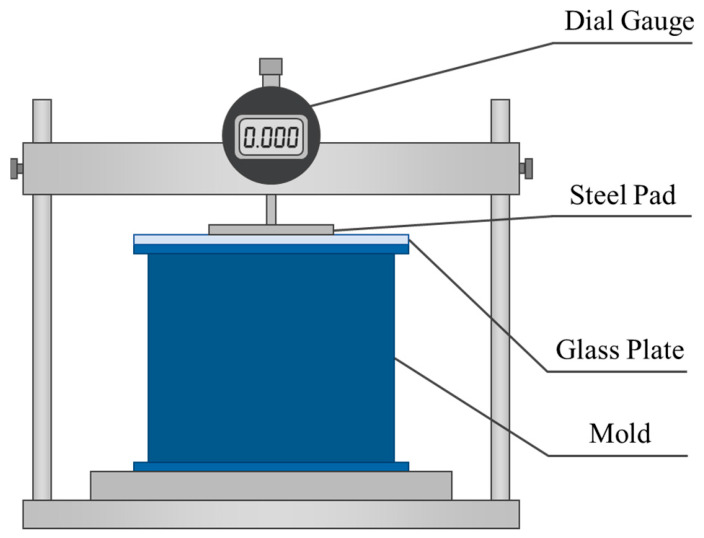
Vertical-expansion-rate measuring device.

The vertical expansion rate of UHPC grout within 24 h was tested by the device shown in Figure 5 in accordance with GB 50119-2013 [22], with one sample for each group. A glass plate (140 mm × 80 mm × 5 mm) and a round steel pad (70 mm in diameter, 5 mm in thickness) were horizontally positioned at the center of the upper surface of a cubic mold (100 mm × 100 mm × 100 mm). Fresh UHPC grout was then cast into the mold until its surface rose approximately 2 mm above the top edge of the mound. A dial indicator was vertically mounted at the center of the steel pad and fixed, and the initial reading *h_0_* was recorded within 30 s. The entire procedure was completed within 3 min. Subsequent readings, *h_t_*, were recorded at 3, 6, 9, 12, and 24 h. All molds were placed at a room temperature of 20 ± 2 °C and relative humidity of 60 ± 5%. The vertical expansion rate was calculated using Equation (1).

(1) $$\varepsilon_{t}=\frac{h_{t}-h_{0}}{h}\times 100$$

where $\varepsilon_{t}$ is the vertical expansion rate, %; $h_{t}$ is the reading at t hours on the dial indicator, mm; $h_{0}$ is the initial reading on the dial indicator, mm; h is the height of the specimen, 100 mm.

#### 2.3.3. Mechanical Properties Tests

The fresh UHPC grout was cast into plastic molds (40 × 40 × 160 mm^3^) and cubic molds (40 × 40 × 40 mm^3^) for flexural and compressive strength tests, respectively. All samples were covered with polyethylene film to prevent moisture loss and stored at ambient laboratory conditions (20 ± 2 °C). Except for the samples requiring testing at 6 h and 1 day, all other samples were demolded 24 h after casting and then cured under standard curing conditions (temperature of 20 ± 2 °C, relative humidity > 95%). The compressive and flexural strength of grout samples were tested after 6 h, 1 day, 3 days, 7 days, and 28 days, based on GB/T 17671-2021 [23], respectively. Six samples for compressive strength and three samples for flexural strength were tested for each group at different curing ages, with the final results calculated as the arithmetic average values.

#### 2.3.4. Hydration Process and Products Analysis

The heat flow of UHPC grout was determined by using an isothermal calorimeter to record the total heat release and the hydration heat evolution from 0 to 72 h. The samples at different ages were immersed in anhydrous ethanol to terminate hydration and subsequently ground into powder for X-ray diffraction (XRD), thermogravimetric analysis (TGA), and differential thermal analysis (DTG) to investigate the hydration process of the UHPC grout. Samples used for XRD analysis were scanned at a rate of 5°/min within a 2*θ* range of 5° to 90°. During TGA and DTG, the powder samples were heated in an air atmosphere from 20 °C to 800 °C at a rate of 10 °C/min and then cooled at 20 °C/min.

#### 2.3.5. Nitrogen Sorption Test

The pore structure of UHPC grout is similar to that of UHPC, and nitrogen sorption analysis is more suitable for characterizing its nanoscale pore size distribution (2–50 nm). Samples for nitrogen sorption tests were crushed into fragments with diameters less than 3 mm after standard curing for 28 days. The samples were immersed in ethanol to terminate the hydration process and then dried at 40 °C for 3 days. The surface area and pore size distribution can be obtained by the BET and BJH methods [24,25].

#### 2.3.6. SEM Test

The microstructure, hydration products, and cracks of the UHPC grout were examined using scanning electron microscopy (SEM). The samples were fragments obtained by crushing UHPC grout specimens after 28 days of standard curing. Before the SEM analysis, the fragments were immersed in ethanol to terminate the hydration process, then dried at 40 °C for 3 days. All samples were gold-coated before testing.

#### 2.3.7. Rapid Chloride Migration Test

Similar to UHPC, UHPC grout exhibits low porosity and high density, which makes it difficult to measure the chloride penetration depth accurately. A conventional Rapid Chloride Migration (RCM) test is insufficient to accurately assess the chloride resistance of UHPC grout. In this study, a combined methodology based on the standard GB/T 50082-2024 [26] and the approach proposed by Shi et al. [27] was adopted. The non-steady-state chloride migration coefficient of UHPC grout was evaluated using an adapted RCM test. To facilitate saturation, cylindrical specimens with a diameter of 100 mm and a height of 30 mm were used in the RCM test. The saturation standing time of the specimens after vacuum saturation before the test was 42 h. After the RCM test, the specimens were split vertically, and a 1 mol/L AgNO_3_ solution was sprayed on the exposed surface. The average chloride penetration depth was then measured. Two samples were tested for each group, and the results were expressed as the arithmetic average. The non-steady-state chloride migration coefficient of the UHPC grout was calculated according to Equation (2).

(2) $$D_{RCM}=\frac{0.0239\times \left(273+T\right)L}{\left(U-2\right)t}(x_{d}-0.0238\sqrt{\frac{\left(273+T\right)Lx_{d}}{U-2}})$$

where $D_{RCM}$ is the non-steady-state chloride migration coefficient, ×10^−12^ m^2^/s; U is the absolute value of the test voltage, V; t is the test duration, h; L is the average thickness of the specimens, mm; $x_{d}$ is the average value of the chloride penetration depth, mm; T is the average initial and final temperature of the anode solution, °C.

## 3. Results and Discussion

### 3.1. Fluidity

**Figure 6:**
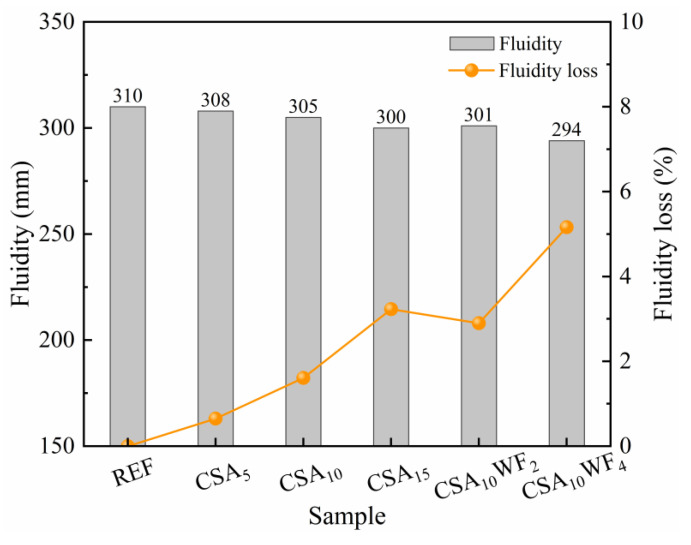
Fluidity of UHPC grout.

As shown in Figure 6, the fluidity of UHPC grout gradually decreased with increasing CSA cement replacement, with a total reduction between 0.6% and 3.2%. The minimum fluidity occurred at a CSA cement content of 15%. CSA cement reacts faster compared to OPC at the early stage of hydration. After contact with water, the formation of hydration products from ye’elimite is influenced by the concentrations of sulfate and lime in the matrix [28]. In the presence of a small amount of calcium hydroxide in the matrix, ye’elimite will form ettringite ($C_{6}A{\bar{S}}_{3}H_{32}$) through Equation (3). In the absence of calcium hydroxide, it reacts through Equations (4) and (5) to produce ettringite, monosulfoaluminate ($C_{4}A\bar{S}H_{12}$), and aluminum hydroxide (AH_3_). Due to the rapid reaction of ye’elimite, a large amount of hydration products are generated and the free water in the matrix is consumed [28], which leads to different degrees of reduction in the fluidity of UHPC grout. However, since CSA cement accounts for less than 15% of the total cement content, the fluidity of UHPC grout is still controlled within an ideal range.

(3) $$C_{4}A_{3}\bar{S}+8C\bar{S}+6CH+90H\to {3C}_{6}A{\bar{S}}_{3}H_{32}$$

(4) $$C_{4}A_{3}\bar{S}+2C\bar{S}+38H\to C_{6}A{\bar{S}}_{3}H_{32}+2{\mathrm{AH}}_{3}$$

(5) $$C_{4}A_{3}\bar{S}+18H\to C_{4}A\bar{S}H_{12}+2{\mathrm{AH}}_{3}$$

After the incorporation of wollastonite fiber, the fluidity of UHPC grout with different wollastonite fiber contents decreased by 2.9% and 5.2%, respectively. The CSA_10_WF_2_ showed a relatively smaller reduction. As the wollastonite fiber content increased, the fluidity of UHPC grout decreased accordingly. This can be attributed to the finer particle size and larger specific surface area of wollastonite fiber compared to cement particles, which increases the water demand of the grout and thus reduces its fluidity [29]. In addition, due to the high aspect ratio of wollastonite fibers, the reduction in fluidity is also likely caused by the interlocking of the needle-like ultrafine wollastonite fibers [15,30].

### 3.2. Vertical Expansion Rate

**Figure 7:**
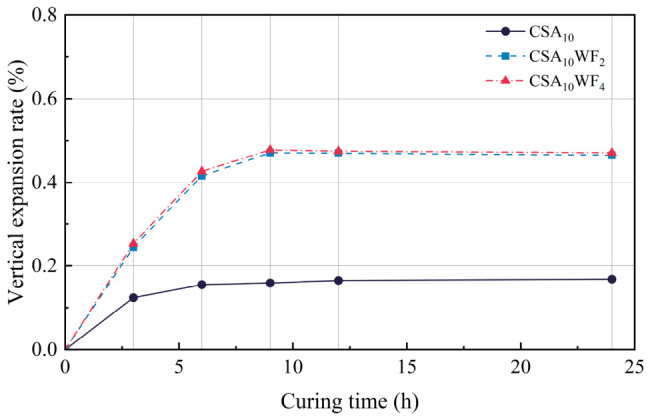
Vertical expansion rate of UHPC grout.

The expansion rate of UHPC grout is shown in Figure 7. The CSA_10_ showed an increased vertical expansion rate, which is attributed to the hydration products of CSA cement in the early stage, such as ettringite and aluminum hydroxide. The formation of ettringite promotes slight expansion in the matrix during early hydration, thereby increasing the 24 h vertical expansion rate of the UHPC grout [31,32]. After the incorporation of wollastonite fibers and UEA, the vertical expansion rate of UHPC grout increased significantly. The differences in expansion among different wollastonite fiber dosages were relatively small, indicating that the UEA contributed more significantly to vertical expansion. The CSA_10_ showed a vertical expansion rate exceeding 0.1% at 3 h, meeting the requirements specified in GB/T 50448-2015 [33] for cement-based grouting materials. With the addition of the UEA, the 3 h expansion rate of UHPC grout ranged from 0.244% to 0.253%, and after 9 h, it reached between 0.471% and 0.478%. A slight decrease in expansion was observed with further curing time due to the shrinkage of the matrix [34].

### 3.3. Mechanical Properties

**Figure 8:**
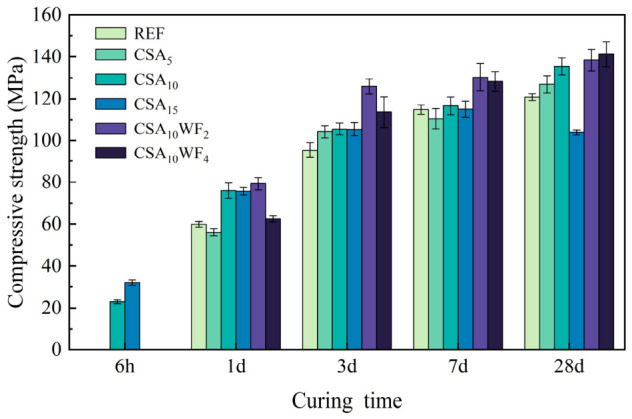
Compressive strength of UHPC grout.

Figure 8 illustrates the development of compressive strength in UHPC grout. The incorporation of CSA cement significantly accelerated the early hydration process. At 6 h, CSA_10_ and CSA_15_ had already achieved compressive strength of 23 MPa and 32.2 MPa, respectively, while the REF and CSA_5_ still were not hardened enough and demolded. After 1 day, the CSA_10_ reached a strength of 75.9 MPa, representing a 26.7% increase compared to the REF group. Additionally, its strength at 3, 7, and 28 days increased by 10.8%, 1.5%, and 12.1%, respectively, compared to the REF group. The improvement in compressive strength for UHPC grout after incorporating CSA cement was primarily concentrated on the first day of hydration. This can be attributed to the high content of ye’elimite in CSA cement, which reacts rapidly during the initial stages of hydration [35].

The early hydration of ye’elimite leads to the rapid formation of ettringite and aluminum hydroxide (AH_3_) gel in the UHPC grout matrix. Concurrently, the hydration of C_3_S and C_3_A also contributes to the generation of C-S-H gel, calcium hydroxide (CH), and additional ettringite through Equations (6) and (7). The formation of CH further promotes Equation (3), resulting in more ettringite, which fills the void in the matrix and enhances the compressive strength of the UHPC grout [28]. Due to differences in mineral composition between CSA cement and OPC, the addition of CSA cement reduces the overall content of C_2_S in the binder system. Since the hydration of C_2_S through Equation (8) is a main contributor to the later stage strength development of UHPC grout, a lower C_2_S content leads to a slower strength development in the later stage. In particular, the CSA_15_ group, which contains a higher proportion of CSA cement, exhibited a strength regression at a curing age of 28 days. The super early hydration reaction of the UHPC-based grout was rapid, and the generated products quickly encapsulated the unhydrated mineral particles. This hindered subsequent hydration reactions and led to defects within the matrix, which may also be the reason for the reduction in strength.

(6) $${2C}_{3}S+6H\to C_{3}S_{2}H_{3}+3CH$$

(7) $$C_{3}A+3C\bar{S}+32H\to C_{6}A{\bar{S}}_{3}H_{32}$$

(8) $${2C}_{2}S+4H\to C_{3}S_{2}H_{3}+3CH$$

After the addition of wollastonite fiber and UEA, the compressive strengths of CSA_10_WF_2_ at four curing ages were 79.2 MPa, 125.9 MPa, 130.3 MPa, and 138.4 MPa, representing increases of 32.2%, 32.1%, 13.5%, and 14.6% compared with the REF group. The significant enhancement in compressive strength is attributed to the filling effect and micro bridge effect of the ultrafine wollastonite fiber, which improves the density and compactness of the cement matrix. However, the reduction in compression strength of CSA_10_WF_4_ at 1 d may due to the high specific surface area of the wollastonite fiber, which tends to accumulate around cement particles and form narrow shells, thereby delaying or weakening the early hydration reactions [36].

**Figure 9:**
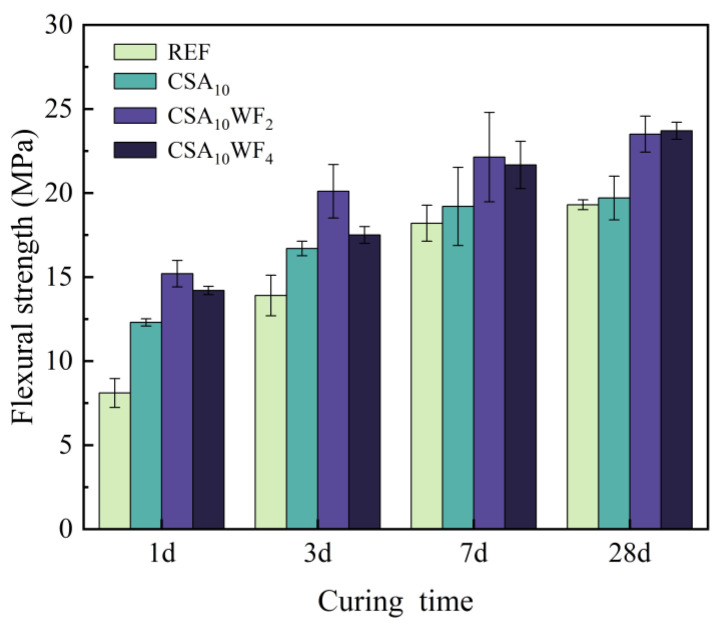
Flexural strength of UHPC grout.

Figure 9 illustrates the flexural strength development of UHPC grout. The trend of flexural strength with increasing CSA cement content closely resembles that of compressive strength. The incorporation of CSA cement significantly improved the flexural strength of CSA_10_ at all curing ages. At 1, 3, 7, and 28 days, the flexural strengths of CSA_10_ reached 12.3 MPa, 16.7 MPa, 19.2 MPa, and 19.7 MPa, corresponding to increases of 51.8%, 20.1%, 5.4%, and 2.1%, respectively, compared with the REF group. The enhancement in flexural strength was most notable at 1 day, which can be attributed to the rapid hydration of ye’elimite in CSA cement, leading to the formation of a large amount of ettringite during the early stage of hydration.

The flexural strength of the CSA_10_WF_2_ at 1, 3, 7, and 28 days were 15.2 MPa, 20.1 MPa, 22.1 MPa, and 23.5 MPa, representing increases of 87.6%, 44.6%, 21.4%, and 21.7%, respectively. Meanwhile, the 28-day flexural strength of CSA_10_WF_4_ reached 23.7 MPa, 22.8% higher than that of the REF group. At early hydration stages, the flexural strength of UHPC grout containing 4 vol% wollastonite fiber was lower than that with 2 vol%. This may be attributed to the higher fiber content partially hindering the early hydration process or weakening the flowability and compactness. UHPC grout with 2 vol% wollastonite fiber exhibited a denser microstructure that enhanced matrix stability and improved flexural strength. As hydration progressed into the middle and later stages, the matrix densities of both mixes became comparable, while the higher fiber content in CSA_10_WF_4_ provided enhanced bridging effects. On the one hand, the wollastonite fiber helped reduce pore volume through the filling effect. On the other hand, the bridging effects and high elastic modulus that wollastonite fiber provides suppressed crack development within the matrix and improved the toughness of the matrix [19,37,38].

### 3.4. Hydration Process and Products

**Figure 10:**
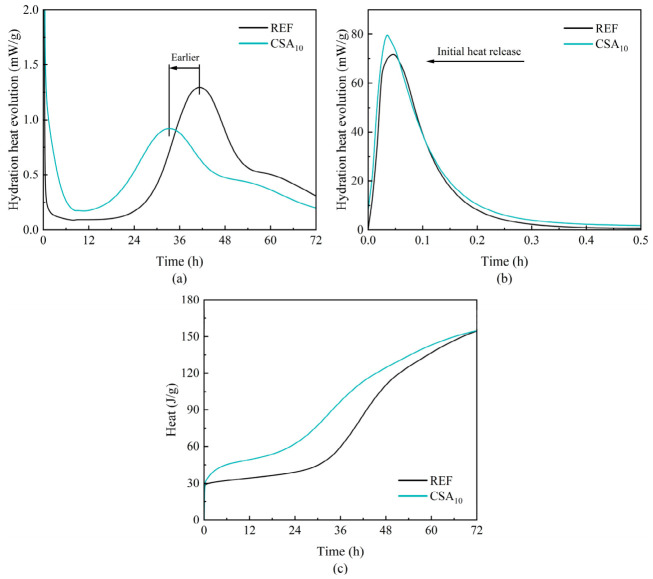
Isothermal calorimetry results: (**a**) hydration heat evolution during 0–72 h, (**b**) hydration heat evolution during 0–0.5 h, (**c**) total heat release during 0–72 h.

Figure 10 presents the hydration heat evolution over different time periods and the total heat release of UHPC grout under 10% CSA cement content. For the CSA_10_, the hydration heat evolution curve remains similar to that of the REF group due to the partial similarity in mineral composition between CSA cement and OPC [39]. The hydration process of UHPC grout typically features an initial rapid and exothermic reaction in the first 0.5 h, followed by an induction period, then an acceleration period and finally a deceleration phase. The initial heat release of the matrix is attributed to wetting and dissolution reactions of the cement particles in contact with free water and the early hydration of ye’elimite [40,41]. The peak observed during the accelerated hydration stage is mainly associated with the hydration of C_3_S [42]. This indicates that CSA can accelerate the hydration process while maintaining the typical hydration sequence [43].

With the addition of CSA cement, the ye’elimite content in the UHPC grout gradually increased. As a result, the initial heat release of UHPC grout was higher, but the heat release during the acceleration stage was reduced. The UHPC grout with CSA cement exhibited a more intense and longer-lasting initial heat release during hydration, mainly due to the presence of ye’elimite [44,45,46] and small amounts of other mineral phases such as mayenite [47]. These mineral phases rapidly hydrate in the presence of soluble sulfates, forming ettringite and various amorphous hydration products [45,46,48]. The lower heat release at the acceleration stage is due to the reduction of C_3_S content in the total cement caused by the addition of CSA cement [49,50].

**Figure 11:**
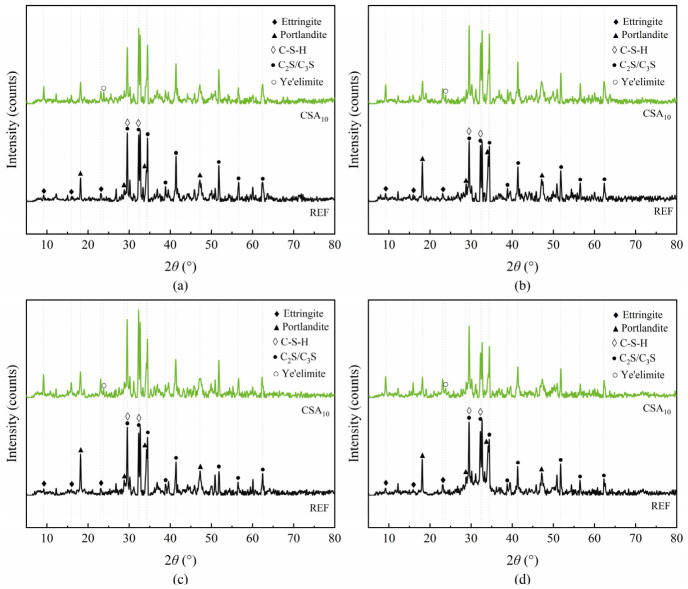
XRD patterns of REF and CSA10 at different ages: (**a**) 1 d, (**b**) 3 d, (**c**) 7 d, (**d**) 28 d.

Figure 11 shows the XRD patterns of UHPC grout with different CSA cement content from 1 day to 28 days. Under the effect of a 10% CSA cement replacement in the CSA_10_ group, the diffraction peaks of the ettringite phase are significantly higher than those of the REF group throughout the entire hydration process. At 1 day, the diffraction peaks of the C-S-H gel phase in the CSA_10_ group were lower than those of the REF group. This is because the main hydration products of CSA cement at the early stage were ettringite and AH_3_, while the OPC in the CSA_10_ group had not yet begun to react extensively. Therefore, the amount of C-S-H gel in CSA_10_ group at this stage was lower than in the REF group. The large amount of ettringite generated filled the pores in the matrix, improving the early strength of the UHPC grout and inducing slight expansion in the matrix. After 3 days, the C_3_S and C_3_A phases in the OPC of CSA_10_ began to hydrate extensively, producing a large quantity of C-S-H gel. However, due to the replacement with CSA cement, the C_2_S content in the binders of CSA_10_ was reduced, which resulted in a relatively slower strength development in the later stage [51,52,53,54].

**Figure 12:**
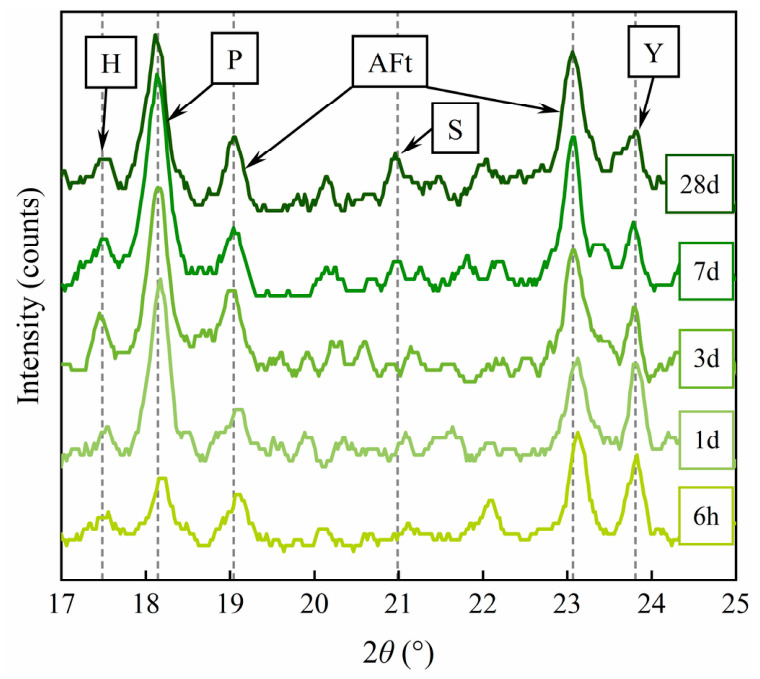
XRD patterns of CSA10 at different ages (P: Portlandite, AFt: ettringite, H: hydrogarnet, S: Strätlingite, Y: ye’elimite).

Figure 12 shows the XRD patterns (17° < 2*θ* < 25°) of the CSA_10_ group from 6 h to 28 days. In the detailed figure, the early hydration behavior of the CSA cement becomes more evident. At 6 h, a considerable amount of ettringite had already formed in the CSA_10_ group. The ye’elimite was significantly consumed within 3 days, and was depleted around 28 days. Meanwhile, the Portlandite phase reached its peak at 7 days and decreased at 28 days, which was due to the pozzolanic reaction occurring in the CSA_10_ matrix. Additionally, the Strätlingite phase (C_2_ASH_8_) and the hydrogarnet phase (C_3_AH_6_) can also be observed in the XRD patterns. Due to the stability of Strätlingite, it contributes to improved mechanical property and durability of the matrix [55,56]. Hydrogarnet is formed from the hydration of C_3_A in Al-rich environments. However, it tends to decompose in Si-rich systems [56], especially in UHPC grout systems where a portion of mineral components remains unreacted. This also explains the reduction in hydrogarnet observed at 28 days.

**Figure 13:**
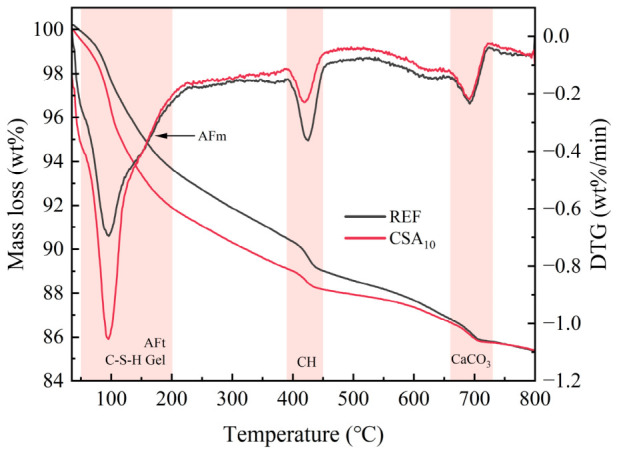
The TG and DTG curves of UHPC grout.

The TG and DTG curves of UHPC grout after 28 days of standard curing are shown in Figure 13. Three distinct peaks can be observed in the DTG curve. The first and most prominent peak appears around 95 °C, which is mainly attributed to the dehydration of ettringite and C-S-H gel [28,44,57]. A significant mass loss also occurs between 120 °C and 180 °C, corresponding to the dehydration of the AFm phase [48,58,59]. The mass loss around 430 °C is associated with the decomposition of CH [41,42]. The content of Portlandite decreases with the incorporation of CSA cement, due to the relatively lower content of C_3_S and C_2_S in the CSA_10_ group. In comparison with the REF group, more ettringite is formed in CSA_10_, while the amount of Portlandite is lower, which helps explain the differences in macroscopic mechanical performance between the CSA_10_ group and the REF group.

### 3.5. Pore Structure

**Figure 14:**
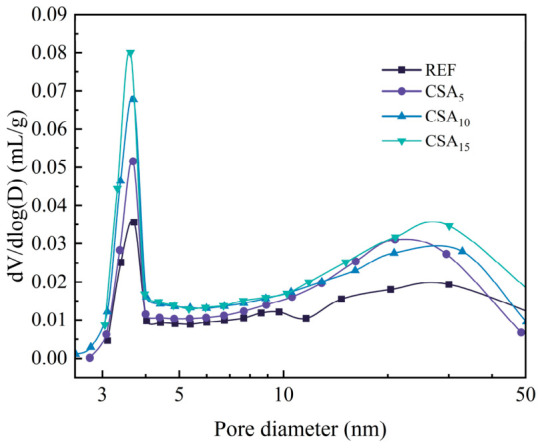
Pore size distribution of UHPC grout.

The pore size distribution of UHPC grout after 28 days of standard curing is shown in Figure 14. The first peak around 3.7 nm gradually shifts to the left, and the peak value increases with the increase in CSA cement content, while the second peak value around 30 nm changes approximately between 0.02 mL/g and 0.04 mL/g. This indicates that the incorporation of CSA cement refined the pore structure of UHPC grout. Due to the volume shrinkage and evaporation of water during the setting and curing process mainly caused by the rapid formation of hydration products, a large number of nanoscale pores are generated, which increases the amount of gel pores in the UHPC grout matrix and enhances the tortuosity of the pore structure [60]. The shift toward finer pores indicate a denser matrix skeleton, which is generally beneficial for improving the effective load-bearing area and stress-transfer continuity within matrix [61]. These gel pores are usually dispersed and non-connected, which have a minimal negative impact on the mechanical load transfer and bearing capacity [60,62].

### 3.6. SEM Analysis

**Figure 15:**
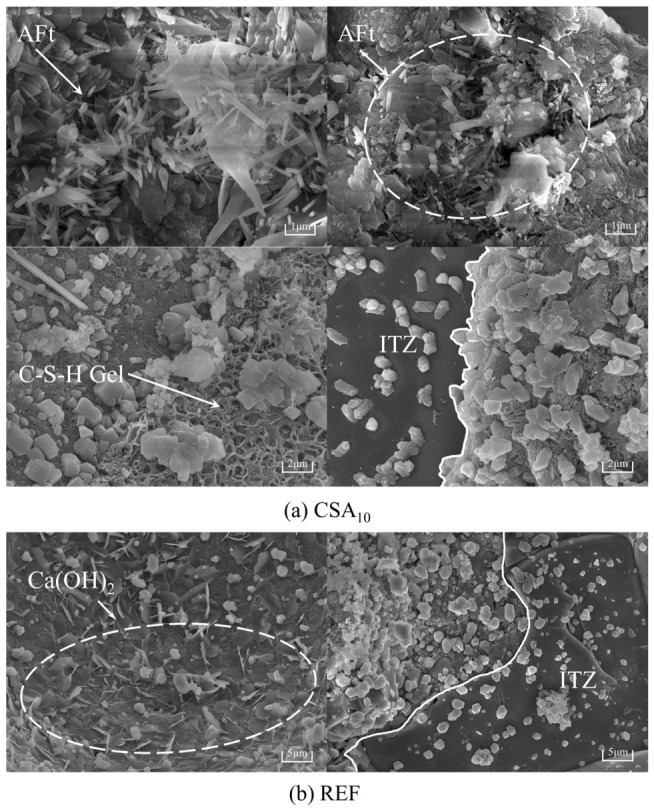
Microstructure of UHPC grout.

Figure 15 presents the SEM images of fractured fragments of CSA_10_ and REF after 28 days of standard curing. In Figure 15a, a large number of needle-like ettringite crystals and honeycomb-like C-S-H gels are observed in the CSA_10_ matrix. These interwoven ettringite crystals, together with C-S-H gel and other hydration products, form a continuous skeleton in the UHPC grout matrix, effectively filling microcracks and pores. This results in a denser microstructure, thereby enhancing the mechanical performance of the UHPC grout. In contrast, Figure 15b shows that the microstructure of the REF group contains concentrated plate-like CH, which is frequently observed in the microstructure of the REF group. This is consistent with the general behavior of modified UHPC systems, in which matrix densification and refined pore structure are closely related to improved mechanical and durability-related performance [63].

As shown in Figure 15a, the interfacial transition zone (ITZ) of CSA_10_ exhibits a well-defined boundary, with a high concentration of hydration product densely packed at the interface. This structure provides strong mechanical interlocking between the matrix and the fine aggregates. The ITZ is notably compact, which contributes to its enhanced strength and is beneficial for improving both the mechanical and chloride resistance performance of the UHPC grout [64]. Since interfacial regions and local defects often act as weak zones in cementitious composites, the denser matrix observed in CSA_10_ may contribute to improved microstructural integrity [64]. By comparison, the ITZ of the REF group in Figure 15b presents an irregular boundary, and the surrounding area mainly consists of unhydrated mineral particles. This kind of loose and discontinuous microstructure weakens the ITZ strength and may affect the durability of the UHPC grout.

### 3.7. Chloride Resistance

**Figure 16:**
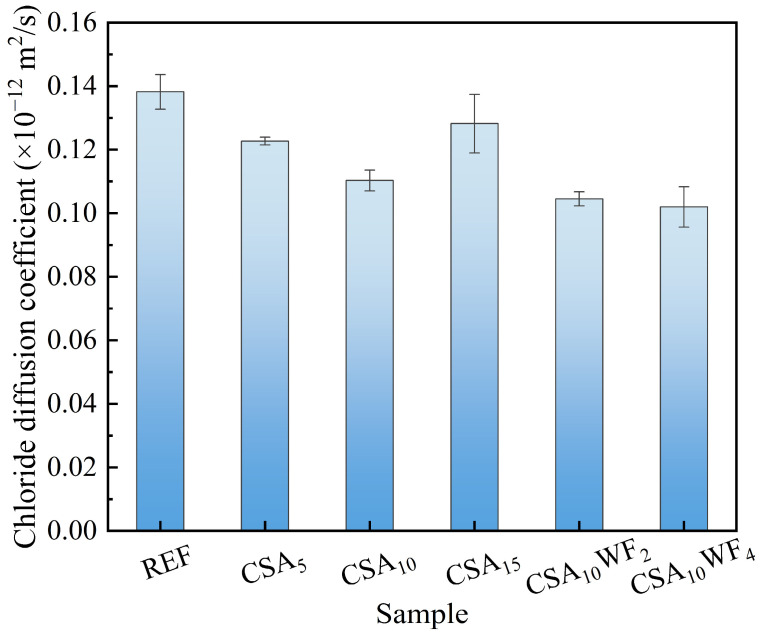
Chloride diffusion coefficient of UHPC grout.

The chloride diffusion coefficient of UHPC grout is shown in Figure 16. The chloride resistance of the UHPC grout improves with increasing CSA cement content, reaching 0.11 × 10^−12^ m^2^/s at CSA_10_, which is 20.2% lower than that in the REF group. However, 15% of CSA cement replacement led to a deterioration in chloride resistance compared to CSA_10_. This might be due to the fact that CSA_15_’s early rapid hydration process resulted in the formation of more harmful pores such as capillary pores, macro pores, and microcracks. The formation of ettringite at the early hydration stage of CSA cement effectively enhances the overall compactness of the matrix. Since the primary ingress pathways for water and chloride in UHPC grout are through pores and microcracks, the enhanced matrix significantly decreases the risk of penetration [65,66]. Pore structure analysis also reveals that although CSA cement incorporation increases the volume of gel pores in some ranges, these additional pores are mostly finer and exhibit lower connectivity. This contributes to increased tortuosity and a stronger capillary blocking effect, thus reducing the continuity of chloride transport channels [67,68].

With the addition of wollastonite fiber and UEA, the chloride resistance of UHPC grout is further enhanced, achieving a minimum chloride diffusion coefficient of 0.102 × 10^−12^ m^2^/s in the CSA_10_WF_4_ group, which is 7.6% lower than that of the CSA_10_ group and corresponds to a 26.2% reduction compared to the REF group. This improvement is attributed to two synergistic mechanisms: on the one hand, the incorporation of wollastonite fibers enhances the internal matrix structure at the microscale. Due to their high aspect ratio and strong interfacial bonding, the wollastonite fibers promote crack-bridging, which helps suppress the initiation and propagation of microcracks, thereby reducing chloride ingress pathways [69]. Additionally, the filler effect of wollastonite fibers increases matrix density and refines the pore structure, reducing the penetration rate of chloride inside the matrix [70]. On the other hand, the optimized early-stage expansion behavior of UHPC grout reduces the microcracks caused by shrinkage, thereby improving matrix compactness. These results indicate that the optimized UHPC grout exhibited improved resistance to chloride migration.

## 4. Conclusions

This research successfully designs rapid-hardening and high-fluidity UHPC grout material and investigates the effects of CSA cement and wollastonite fiber contents on its key properties. The main conclusions can be drawn:

1. The incorporation of CSA cement as a partial replacement for OPC achieves a rapid-hardening UHPC grout, with only a minor reduction in fluidity. The 10% CSA cement contributes to compressive strength of 23 MPa at 6 h. Furthermore, the compressive strengths at 1, 3, 7, and 28 days increased to 75.9 MPa, 105.6 MPa, 116.6 MPa, and 135.4 MPa, respectively, representing improvements of 26.7%, 10.8%, 1.5%, and 12.1% compared with the reference grout.
2. The rapid hydration in CSA cement in the first day generated a large amount of ettringite, significantly enhancing the matrix strength and inducing expansion to mitigate shrinkage in the UHPC grout. However, a higher CSA cement content resulted in slower strength development at later ages, and even strength retrogression was observed.
3. The UHPC grout with CSA cement exhibited increased gel pores in the 2–50 nm range but slightly improved chloride resistance. The high specific surface area and aspect ratio of wollastonite fibers, together with UEA, increased water demand and reduced UHPC grout fluidity by 2.9–5.2%. The incorporation of wollastonite fibers and UEA enhanced the early-age vertical micro expansion rate and compressive and flexural strengths of UHPC grout.
4. In this study, 10% CSA cement and 2% wollastonite fibers are recommended for designing rapid-hardening and high-fluidity UHPC-based grout by comprehensively considering fresh and hardened properties under the tested conditions. The synergistic effect of CSA cement and wollastonite fibers not only enhances mechanical properties of UHPC grout, particularly at an early age, but also maintains its high fluidity. These combined properties significantly broaden the application potential of UHPC grout in projects requiring high-fluidity, high-strength, and high-chloride-resistant materials.

Although this study confirms the potential of CSA cement and wollastonite fibers for developing rapid-hardening UHPC grout with high initial spread flow, some limitations remain. The fresh-state performance, long-term dimensional stability, durability under coupled exposure conditions, and field grouting applicability require further investigation.

## Figures and Tables

**Table 1:** Chemical composition of powder materials (wt%).

Substance	OPC	CSA	SF	Slag	WF
CaO	61.06	45.54	0.02	35.17	49.83
SiO_2_	22.53	6.90	96.82	35.35	48.75
Al_2_O_3_	6.91	20.26	0.23	17.75	0.24
Fe_2_O_3_	2.68	1.54	0.26	0.41	0.37
K_2_O	0.68	0.35	0.02	0.40	0.04
Na_2_O	0.29	-	0.04	0.42	0.03
SO_3_	2.16	24.18	0.03	2.54	-
MgO	3.16	-	-	6.97	0.69
TiO_2_	0.44	1.00	-	0.54	-
Mn_2_O_3_	0.09	0.04	-	0.45	-
P_2_O_5_	-	0.12	-	-	0.05
ZrO_2_	-	0.07	2.58	-	-

**Table 2:** Proportions of UHPC grouts (kg/m^3^).

No.	OPC	CSA	SF	Slag	QS	W	SP	UEA	Steel Fiber (% vol)	WF (% vol)
REF	780	-	60	360	960	216	7.2	-	2	-
CSA_5_	741	39	60	360	960	216	7.2	-	2	-
CSA_10_	702	78	60	360	960	216	7.2	-	2	-
CSA_15_	663	117	60	360	960	216	7.2	-	2	-
CSA_10_WF_2_	702	78	60	360	960	216	7.2	36	2	2
CSA_10_WF_4_	702	78	60	360	960	216	7.2	36	2	4

## Data Availability

The original contributions presented in this study are included in the article. Further inquiries can be directed to the corresponding author.
